# Calpain-mediated vimentin cleavage occurs upstream of MT1-MMP membrane translocation to facilitate endothelial sprout initiation

**DOI:** 10.1007/s10456-012-9262-4

**Published:** 2012-03-11

**Authors:** Hyeong-Il Kwak, Hojin Kang, Jui M. Dave, E. Adriana Mendoza, Shih-Chi Su, Steve A. Maxwell, Kayla J. Bayless

**Affiliations:** Department of Molecular and Cellular Medicine, Texas A&M Health Science Center, College Station, TX 77843-1114 USA

**Keywords:** Sprout initiation, Endothelial, Collagen, Three-dimensional matrix, Proteolysis

## Abstract

Endothelial cells normally line the vasculature and remain quiescent. However, these cells can be rapidly stimulated to undergo morphogenesis and initiate new blood vessel formation given the proper cues. This study reports a new mechanism for initiating angiogenic sprout formation that involves vimentin, the major intermediate filament protein in endothelial cells. Initial studies confirmed vimentin was required for sphingosine 1-phosphate (S1P)- and growth factor (GF)-induced endothelial cell invasion, and vimentin was cleaved by calpains during invasion. Calpains were predominantly activated by GF and were required for sprout initiation. Because others have reported membrane type 1-matrix metalloproteinase (MT1-MMP) is required for endothelial sprouting responses, we tested whether vimentin and calpain acted upstream of MT1-MMP. Both calpain and vimentin were required for successful MT1-MMP membrane translocation, which was stimulated by S1P. In addition, vimentin complexed with MT1-MMP in a manner that required both the cytoplasmic domain of MT1-MMP and calpain activation, which increased the soluble pool of vimentin in endothelial cells. Altogether, these data indicate that pro-angiogenic signals converge to activate calpain-dependent vimentin cleavage and increase vimentin solubility, which act upstream to facilitate MT1-MMP membrane translocation, resulting in successful endothelial sprout formation in three-dimensional collagen matrices. These findings help explain why S1P and GF synergize to stimulate robust sprouting in 3D collagen matrices.

**Electronic supplementary material:**

The online version of this article (doi:10.1007/s10456-012-9262-4) contains supplementary material, which is available to authorized users.

## Introduction

Angiogenesis is the formation of new blood vessels from pre-existing structures and is a key step in development, wound healing and pathological events [1, 2]. While the endothelium is normally quiescent and exhibits infrequent turnover, endothelial cells (ECs) that line the vascular system must respond rapidly to external cues to initiate angiogenesis and extend new sprouts into the surrounding extracellular matrix. Exogenous extracellular pro-angiogenic factors such as vascular endothelial growth factor (VEGF), basic fibroblast growth factor (bFGF) and sphingosine 1-phosphate (S1P), potently stimulate new blood vessel growth [3–5]. Thus, in response to various pro-angiogenic cues, ECs initiate the formation of new vascular networks. However, the molecular mechanisms that mediate angiogenic sprouting are not completely understood.

Here, we investigate the intermediate filament protein vimentin as a regulator of angiogenic sprouting. Intermediate filament proteins were initially thought to function solely for mechanical stabilization of cells but are more recently being recognized as regulators of signal transduction [6]. Endothelial cells express the intermediate filament vimentin [7], and although no overt phenotypic alterations were observed in the original report of vimentin null animals [8], subsequent studies revealed defects in endothelial barrier function [9]. The lack of an overt phenotype in vimentin null animals [8], combined with a multitude of evidence that vimentin is involved in signal transduction in isolated cell systems [6] has created a conundrum in the field with respect to the function of vimentin [7]. Eckes et al. [10] reported that vimentin null mice exhibit a lag in granulation tissue formation, a process that involves extensive angiogenic sprouting. Further, vimentin null mice have reduced corneal neovascularization [10] and hypoxia-induced retinal neovascularization responses [11]. Although these anecdotal examples suggest intermediate filaments are necessary for angiogenic responses, the underlying mechanism remains unclear and further investigation is warranted.

Calpains are intracellular calcium-activated cysteine proteases and are viable candidates for controlling the transition from adherent to invasive ECs. Calpains regulate cell migration on two-dimensional substrates by cleavage of the actin regulatory proteins talin, vinculin, paxillin, focal adhesion kinase and cortactin [12–14]. Calpains are required for membrane protrusion, focal contact dissolution, cell membrane release during cell spreading, and invadopodia formation [13, 15–17]. Calpains can also cleave the N-terminus of the intermediate filament protein, vimentin [18, 19], rendering vimentin incapable of forming insoluble, polymerized intermediate filaments [20]. Pro-angiogenic factors, including VEGF, activate calpains [21–23], and calpain inhibitors block angiogenesis in vivo [22, 24, 25]. These studies provide a supportive platform to investigate a functional requirement for calpains in initiating angiogenic sprouting events and determine potential downstream intracellular signals that result from calpain activation.

Membrane-type matrix metalloproteinases (MT-MMPs) coordinate with growth factors (GF) and integrins to direct angiogenic sprouting and lumen formation [26–31]. While MT1-MMP is required for vessel outgrowth and lumen formation, the precise intracellular molecular events that control MT1-MMP activation and membrane translocation following stimulation with pro-angiogenic factors are not completely defined. In this study, we uncover a new pathway where calpain cleavage of vimentin facilitates MT1-MMP membrane localization to initiate angiogenesis in primary ECs. Calpain activation and increased vimentin solubility are key molecular events that support pro-angiogenic factor-stimulated MT1-MMP-dependent sprouting. This study is the first to demonstrate an endothelial-specific requirement for the intermediate filament, vimentin, in directing angiogenic events. Our data provide new information about the molecular signals responsible for endothelial cell sprouting and have implications for a general role of this pathway in regulating cell invasion.

## Materials and methods

### Reagents

Human umbilical vein ECs (Lonza) were maintained as previously described [32] and used at passages 3–6 for all invasion and wound healing studies. Calpain inhibitor III (Z-Val-Phe-H; MDL 28170) was purchased from EMD Biosciences. All other reagents were from Sigma-Aldrich unless indicated.

### Expression vector construction

Human MT1-MMP was amplified from human umbilical vein endothelial cell cDNA generated in the laboratory [33] and cloned into EGFP-N2 vector (Clontech). An MT1-MMP-RFP construct was also constructed using the pTagRFP-N expression vector (Axxora, San Diego, CA). Human TIMP-1 and TIMP-3 were amplified from human placental cDNA (Clontech) and inserted into the pIEX-5 vector (Novagen) to generate C-terminal S- and His-tags. Full length MT1-MMP (Full) and a cytoplasmic tail deletion mutant (ΔCT) were also cloned into pIEX-5. Inserts containing a C-terminal S-tag were cloned into pENTR4 vector and recombined into the pLenti6/V5 Dest vector (Invitrogen) according to manufacturer’s instructions. All constructs were confirmed by sequence analysis and expression in 293FT cells.

### Endothelial cell invasion assay

Assays to study endothelial invasion responses were established and quantified as previously described [32, 33]. Collagen matrices were prepared containing 1 μM S1P (Avanti Polar Lipids). After thorough mixing, collagen was added at 28 μl per well in 4.5 mm diameter 96-well plates (Costar). The collagen was allowed to equilibrate for 30 min at 37°C in a CO_2_ incubator before adding cells (40,000 per well) resuspended in 100 μl of medium containing reduced serum II (RSII), recombinant human VEGF and bFGF and ascorbic acid. Cells were allowed to invade for 24 h. Culture medium was removed and collagen matrices containing invading ECs were fixed in 3% glutaraldehyde in PBS overnight. Matrices were stained with 0.1% toluidine blue in 30% methanol for 10 min prior to destaining with water.

### Transient transfection on coverslips

One microgram of endotoxin-free plasmid DNA in 50 μl of Opti-MEM (OMEM, Invitrogen) was mixed with 3 μl of Lipofectamine 2000 (Invitrogen) in 50 μl OMEM, incubated for 20 min at room temperature and then added to 400 μl of culture medium containing 10^5^ ECs. Mixtures were added to 12 mm circular cover slips pre-coated with 20 μg/ml collagen type I and allowed to attach for 1 h. Cells were rinsed with M199 and maintained overnight in antibiotic-free growth media. Expression of EGFP fusion proteins was checked by fluorescence microscopy and confirmed by Western blot analysis.

### Quantifying invasion responses

Invasion densities were quantified by counting fixed cultures under transmitted light using an Olympus CK2 inverted microscope equipped with eyepieces with a 10 × 10 ocular grid. For each condition, four random fields were selected and the number of invading cells per high power field (HPF) was counted manually at 20× magnification (approximately 0.25 mm^2^). Data are reported as mean numbers of invading cells per HPF (±SD). Invasion length and lumen diameter were measured using digital images taken from a side view of cultures at 4× magnification. For each condition, cells (n = 100) were measured and average invasion distance in micrometers is presented (±SD).

### Calpain activity assay

HUVEC were plated at 50–80% confluence in a 96 well plate and incubated with M199 medium containing RSII for 8 h. The cells were pre-treated with calpain inhibitors or DMSO for 1 h and then loaded with 30 μM of the calpain substrate tBoc-LM-CMAC (Invitrogen). The cells were treated with or without S1P, GF or S1P + GF for 30 min, and imaged using a Nikon TE-2000 fluorescent microscope (excitation 329 nm, emission 409 nm). Fluorescence intensity was measured using Image J software. A second calpain activity assay was performed using Calpain-Glo™ Protease Assay Kit (Promega). Cells from 3D collagen matrices were collected in lysis buffer (0.9% Triton X-100, 100 μmol/l PMSF and 20 μg/ml aprotinin in PBS). Freshly prepared Calpain-Glo™ Reagent was mixed with samples (1:1) and added to each well of a white 96-well plate containing 40 μl of blank or test sample and incubated at room temperature for 15 min before analyzing on a LumiCount luminometer. Experiments were performed three times in triplicate wells. One well represents six individual collagen matrices from 3D invading cultures.

### MT1-MMP activity assay

MT1-MMP activity assays were performed using SensoLyte™ 520 MMP-14 Assay Kit (Anaspec). Endothelial cells were transduced with lentiviruses expressing GFP, TIMP-1, and TIMP-3 and placed on 3D collagen matrices (six matrices per treatment per time point). Samples were collected at 6 h of invasion, lysed at 4°C for 10 min, and centrifuged for 10 min at 2,500×*g* at 4°C. Supernatants were collected and stored at −80°C until use. Working solutions were prepared as directed with MT1-MMP substrate. Test reactions along with positive and negative controls (40 μl) were combined with the substrate solution. Fluorescence intensity at Ex/Em = 490/520 nm was read and continuously recorded every 5 min for 60 min. Experiments were performed three times in triplicate wells. Average values were recorded and plotted with standard deviation.

### Generation and transduction of TIMP-1 and TIMP-3 lentivirus

Lentiviruses were generated as described [33]. Seventy-two hours after viral transduction, cells were given fresh growth media and 2 μg/ml blasticidin for 14 days. Confluent cultures were tested in 3D invasion assays [33]. Successful expression of recombinant proteins was confirmed by Western blot analysis using S-protein-HRP conjugate (Novagen) or antisera specific to proteins of interest.

### Immunofluorescence

Coverslips were fixed in freshly prepared 4% paraformaldehyde in PBS for 10 min. Each was rinsed three times in Tris/Glycine buffer (0.3% Tris, 1.5% Glycine) and permeabilized with 0.5% Triton X-100 in PBS for 30 min with gentle agitation. Samples were blocked in buffer containing 0.5% TX-100, 1% BSA, and 1% serum overnight at 4°C. Primary antibodies were added in blocking buffer (1:100) for 2 h at room temperature. After three washes (5 min each) in 0.1% TX-100 in PBS, Alexa-488- or -594-conjugated secondary antibodies (Molecular Probes) were added (1:200) in blocking buffer for 1 h. After washing, samples were mounted and imaged using a Nikon TE-2000 fluorescent microscope equipped with appropriate filters.

### Immunofluorescence analyses

To quantify MT1-MMP-GFP localization at the cell periphery, outlined section of cells were manually traced in Adobe Photoshop and the pixel intensity inside a 10-pixel-wide outline of the cell was quantified in Image J. To avoid measuring fluorescent intensity from perinuclear staining, any perinuclear staining that entered the outline was excluded from the analysis. A cell fluorescence intensity histogram was normalized by setting the darkest cytoplasmic region in the cell to a pixel intensity of zero. The extent of MT1-MMP-GFP localization to the cell periphery was defined as the average pixel intensity within the analysis region. The quantification of MT1-MMP-GFP localization to the cell periphery was obtained from three individual experiments (n = 25 cells).

### Immunoprecipitations

Subconfluent ECs (1 × 10^6^) were harvested and washed twice with 10 ml of ice-cold PBS. Cell pellets were lysed in 1 ml of cold lysis buffer [50 mmol/l Tris–HCl, pH 7.5, 150 mmol/l NaCl, 1% NP40, 0.5% Sodium Deoxycholate, 1 mmol/l PMSF, and 1× protease inhibitor cocktail (Roche)] and incubated for 20 min on ice with occasional mixing. Cell lysates were centrifuged at 14,000 rpm for 15 min at 4°C and supernatants were collected and incubated with protein G-agarose (Pierce) at 4°C with agitation for 1 h before supernatants were incubated with 2 μg/ml of antisera directed to vimentin (V9, Santa Cruz Biotechnology) or MT1-MMP (ab38971, Abcam) for 18 h at 4°C with agitation. Protein G-agarose was added for 2 h at 4°C. Pellets were washed five times with 1 ml lysis buffer without protease inhibitors and analyzed by Western blotting. For 3D immunoprecipitations, HUVECs (40,000/well) were seeded on collagen matrices in 96 well plates and allowed to invade for 3 h. Cells were washed with ice-cold PBS and extracted in ice-cold lysis buffer (40 gels/group).

### Generation of stable knockdown cell lines using shRNA

Lentiviral vectors specific for calpain 1 (#SHCLNG-NM005186), calpain 2 (#SHCLNG-NM001748) and vimentin (#SHCLNG-NM3380) shRNA were purchased from Sigma-Aldrich. Lentiviral particles were generated by combining 1.5 μg of backbone shRNA lentiviral plasmid with 4.5 μg of VIRAPOWER packaging mix (Invitrogen) into 293FT cells, using Lipofectamine 2000™ transfection reagent (Invitrogen) in T25 flasks. Viral supernatants were harvested at 72 h, centrifuged at 300×*g* for 5 min, filtered through a 0.45 μm filter (Millipore) and incubated with 0.4 × 10^6^ HUVEC (passage 2–3) and polybrene (12 μg/ml). Four hours after viral transduction, cells were given fresh growth media. Stable transfectants were selected in the presence of 0.2 μg/ml puromycin for 2 weeks prior to testing in invasion assays.

### Cell surface biotinylation

Cell surface biotinylation was conducted as described by Stack et al. [34]. Cells were grown to confluence in 6-well plates, washed twice with 2 ml ice-cold PBS containing 2 mM Ca^2+^ and 1 mM Mg^2+^, and incubated at 4°C with gentle shaking for 30 min with 0.5 mg/ml cell-impermeable EZ-Link sulfo-NHS-LC-LC-biotin [sulfosuccinimidyl-6′-(biotinamido)-6-hexanamido hexanoate] (Pierce) in 1 ml ice-cold PBS, followed by washing twice with ice-cold PBS and once with 100 mmol/l glycine to quench free biotin. Cells were detached by scraping, lysed in lysis buffer (50 mM Tris, pH 7.4, 150 mM NaCl, 0.5% sodium deoxycholate, 1% NP40) with proteinase inhibitors (Roche), and clarified by centrifugation. To isolate biotinylated cell surface proteins, equal amounts of protein from each sample were incubated with streptavidin beads at 4°C for 14 h, followed by three washes with 0.5 ml lysis buffer before centrifugation. After boiling in sample buffer to dissociate streptavidin bead-biotin complexes, the biotin-labeled samples were analyzed by 9% SDS-PAGE and immunoblotted for MT1-MMP, vimentin, and GAPDH.

### Isolation of soluble vimentin fractions

ECs seeded on 3D collagen matrices were allowed to invade for 6 h. Six collagen matrices per treatment were washed with cold PBS containing cations. 40 μl of ice-cold solubilization buffer (1% NP40, 0.5% sodium deoxycholate, 1× protease inhibitor cocktail (Roche), and 1× HALT protease inhibitor) was added to each well. Plates were placed on ice for 20 min and rotated at 50 rpm. Supernatants from each well were collected, pooled, and spun at 12,000×*g* for 10 min at 4°C. Cleared supernatants (200 μl) were combined with 100 μl of 3× sample buffer and boiled for 5 min before analysis using Western blot.

### Preparation of membrane fractions from 3D collagen matrices

Invading EC cultures were established without and with S1P + GF for 3 h. Sixty collagen matrices were collected in 5 ml homogenization buffer [20 mM HEPES (pH 7.4), 1 mM EDTA, 250 mM sucrose, 20 mM NaCl, 1.5 mM MgCl_2_, Protease inhibitor cocktail (Roche), phenylmethylsulfonyl fluoride (2 mM), 1 mmol/l Mercaptoethanol, 0.1 mg/ml collagenase] and digested at 4°C. Cells were disrupted by 10 strokes through a 27 gauge needle and homogenates were centrifuged at 6,300×*g* for 5 min to remove unbroken cells. The supernatant was centrifuged at 150,000×*g* for 30 min. Pellets were reconstituted in 300 μl Laemmli sample buffer and boiled for 5 min.

## Results

### Vimentin is required for endothelial cell invasion

**Fig. 1:**
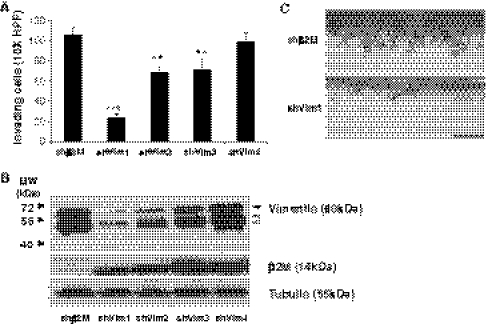
Vimentin knockdown interfered with invasion responses. **a** Quantification of invasion density resulting from shRNA-mediated knockdown of beta 2 microglobulin (shβ2M) or vimentin (shVim). Four independent sequences are shown for vimentin knockdown (shVim1-4). Cells expressing indicated shRNA sequences were allowed to invade in the presence of S1P, VEGF, and bFGF, as previously described [32]. Cultures were fixed at 16 h of invasion and a representative experiment (n = 4) is shown. Data presented are average values from five 1 mm^2^ fields (±SD). ***P < 0.001, **P < 0.01 versus shβ2M, Student’s *t* test. **b** Western blot analyses of whole cell lysates of invading cells (16 h) using vimentin-, beta 2 microglobulin (β2M)-, and tubulin-specific antisera. Open arrowheads indicate vimentin cleavage products. **c** Representative photographs from a side view illustrate invasion responses observed with control and vimentin knockdown cells. Scale bar, 100 μm

To investigate the role of intermediate filament proteins in angiogenic sprouting, we first tested whether vimentin was required for S1P- and GF-induced endothelial cell invasion in 3D collagen matrices. Four individual shRNA sequences directed to vimentin (shVim 1-4) were introduced using lentiviral transduction and compared to β2 microglobulin (shβ2M) shRNA as a negative control. Knockdown of vimentin significantly interfered with endothelial cell invasion responses to SIP and GF (Fig. 1a), and invasion directly correlated with vimentin expression levels (Fig. 1b). We also observed cleavage fragments of vimentin (indicated by open arrowheads, Fig. 1b). Photographs of invading cultures in control (shβ2M) and shVim1-expressing cells are shown in Fig. 1c. The results demonstrate vimentin is required for and cleaved during primary human endothelial cell invasion of collagen matrices stimulated by S1P and GF.

### Vimentin cleavage is calpain-dependent, and GF predominantly activate calpains

**Fig. 2:**
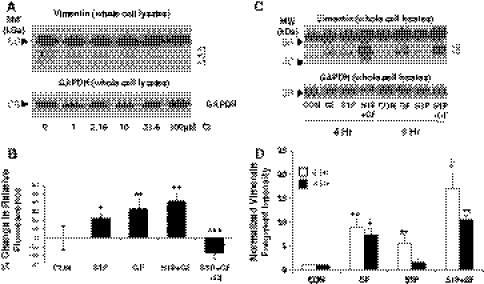
Calpains are activated by growth factors and result in vimentin cleavage. **a** Invading cultures were treated with indicated doses of CI and allowed to invade collagen matrices for 22 h in the presence of S1P and GF. Whole cell extracts were subjected to Western blotting and probed with antisera directed to vimentin. Blots were stripped and re-probed with GAPDH- specific antisera. Results are representative of three independent experiments. **b** ECs were plated at 80% confluence in a 96 well plate in M199 medium containing RSII for 8 h. The cells were pre-treated with 31.6 μM CI or DMSO (CON) for 1 h and then loaded with 30 μM of the calpain substrate tBoc-LM-CMAC. Cells were treated without (CON) or with S1P (1 μM), GF (40 ng/ml VEGF and bFGF) or S1P + GF for 30 min and imaged. Calpain activity was quantified as indicated in the “Materials and methods” section. Results are representative of four independent experiments. Data shown are average values ± SD. *P < 0.05, and **P < 0.01 compared to Control, ***P < 0.001 compared to S1P + GF by Student’s *t* test. **c** Endothelial cells were seeded on 3D collagen matrices and allowed to invade for 4 or 6 h. Whole cell lysates were prepared and analyzed by Western blotting with antisera directed to vimentin and GAPDH control. **d** Quantification of intensities of vimentin cleavage products with treatment conditions. Data are derived by averaging band intensities from three independent experiments. **P < 0.01 versus CON; ^‡^ *P* < 0.05 versus all other treatments by Student’s *t* test

Calpains are intracellular cysteine proteases that can cleave vimentin [18, 19]. To determine whether vimentin cleavage during endothelial cell invasion was mediated by calpains, lysates were prepared from invading cultures treated with a specific pharmacological calpain antagonist, calpain inhibitor-III (CI), which targets the active site thiol in calpains [35]. Inhibition of calpains using CI dose-dependently blocked vimentin cleavage during invasion (Fig. 2a). These data show that calpain-dependent vimentin cleavage occurs during endothelial cell invasion. In the 3D endothelial sprouting system, S1P combines with GF to stimulate invasion (Supplemental Figure 1). Because calpains cleaved vimentin during sprouting responses, we hypothesized that S1P and GF would activate calpains. To measure calpain activation, endothelial cell monolayers were loaded with 30 μM CMAC, *t*-BOC-Leu-Met, a reliable indicator of calpain activation in live cells [36]. Cells were treated with nothing (CON), S1P, GF, and S1P + GF. A separate group was pre-treated with CI before the addition of S1P and GF (S1P + GF + CI). Images were collected 30 min after treatment (Fig. 2b) and reveal that S1P increased calpain activation relative to control, but GF and S1P + GF were more effective at increasing calpain activation compared to CON, or S1P. Signal intensity in cells treated with S1P + GF + CI was significantly decreased compared to control, indicating CI treatment significantly reduced calpain activation. Although similar experiments for quantifying calpain activity were conducted with 3D cultures, this method was not compatible with polymerized collagen matrices (data not shown). To test whether SIP or GF activated calpain in 3D cultures, an alternative approach was used with 6 h invading ECs that utilized a luminometric substrate that selectively detected activated calpains. S1P increased calpain activity compared to control (Supplemental Figure 2A). Calpain activity was further enhanced by GF, and combining S1P + GF resulted in maximal calpain activation. Also, CI blocked calpain activation (Supplemental Figure 2B), demonstrating efficacy of this compound against calpains. These results suggest that GF are more effective than S1P at increasing calpain activation in ECs. To test whether vimentin cleavage occurs in response to S1P or GF in invading ECs, whole cell extracts were collected after 6 h of invasion. At 4 h, GF appear to induce slightly more vimentin cleavage than S1P (Fig. 2c), and this is more obvious at 6 h of invasion, which is when sprout initiation begins [33]. Data were quantified in Fig. 2d and reveal GF are significantly more effective at inducing vimentin cleavage in ECs seeded on 3D collagen matrices than S1P, agreeing with results in Fig. 2b and Supplemental Figure 2.

### Calpain activation partially regulates endothelial cell invasion

**Fig. 3:**
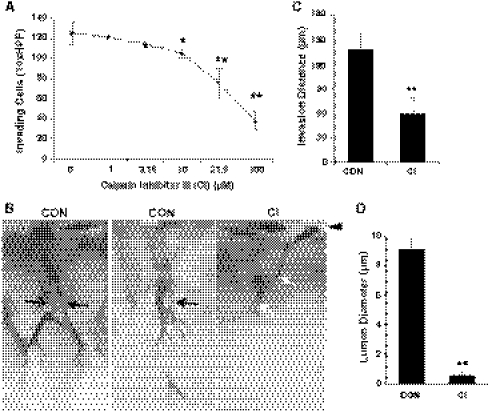
Endothelial cell invasion stimulated by S1P and GF requires calpain activation. **a** Invasion experiments were established by pre-incubating cells with CI at the concentrations indicated for 30 min prior to seeding on collagen matrices. Cells were allowed to invade for 22 h. Data represent average numbers of invading cells per standardized field ± SD (n = 4). **b** Representative photographs of a *side view* of invading cells from control (CON) and 100 μM CI treatment. *Arrowhead* indicates original monolayer. *Black arrows* indicate a lumen and *white arrows* indicate altered structures observed with CI treatment. **c** Quantification of average invasion distances (in microns) from monolayer to the tip of invading structures control (CON) and CI treated group (n = 100 cells). **d** Quantification of lumen diameter (in microns) of invading structures in control (CON) and 100 μM CI treated group (n = 100 cells). Results are representative of three independent experiments. Data shown in **a**, **c**, and **d** are average values ± SD. *P < 0.05, **P < 0.01 versus control by Student’s *t* test

To test for a functional role for calpains in endothelial cell invasion responses, cells were treated with CI. Quantification of invasion responses revealed a dose-dependent inhibition of invasion by CI (Fig. 3a), associating calpain activation, vimentin cleavage and invasion. No adverse effects on cell monolayers or evidence of compromised cell viability were observed with calpain inhibition, and vehicle had no effect on invasion (data not shown). Sprout morphology of invading cells was significantly altered in the presence of CI (Fig. 3b). Representative side views of invading structures (Fig. 3b) shows control cultures are multicellular and contain lumens (indicated by black arrows), while those treated with CI exhibited thin sprouts and failed to form multicellular structures surrounding a lumen (Fig. 3b, white arrows). We observed a marked decrease in sprout length, reported as invasion distance (Fig. 3c) and nearly complete blockade of lumen formation (Fig. 3d). These findings support that calpains are required for endothelial cell invasion responses.

**Fig. 4:**
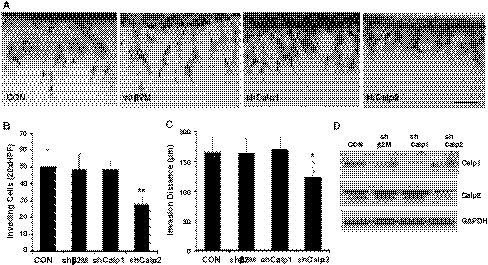
Calpain knockdown significantly reduced EC invasion. ECs were not treated (CON) or transduced with lentiviruses delivering shRNA directed to beta 2 microglobulin (shβ2M; negative control), calpain 1 (shCalp1) and calpain 2 (shCalp2). Stable cell lines were selected with puromycin (0.2 μg/ml) for 2 weeks prior to testing in invasion assays. **a** Representative photographs of a *side view* of invading cells from each treatment group. *Bar* = 100 μm. **b** Quantification of the average number of invading cells per 0.25 mm^2^ field (n = 4 fields) from a representative experiment (n = 4); **P < 0.01 versus control, Student’s *t* test. **c** Quantification of the average invasion distances (in μm) recorded from monolayer to leading edge of invading structures (n = 100 cells). Data shown are average values ± SD. *P < 0.05 versus control, Student’s *t* test. **d** Western blot analyses of whole cell lysates from invading cultures (24 h) probed with calpain 1- (Calp 1), calpain 2- (Calp 2) and GAPDH-specific antisera

To confirm pharmacological studies, recombinant lentiviruses delivering short hairpin RNA (shRNA) were generated to silence beta 2 microglobulin (shβ2M), calpain 1 (shCalp1) and calpain 2 (shCalp2). A side view of invasion responses is shown in Fig. 4a. Cell lines stably expressing shRNAs exhibited no changes in morphology compared to wild-type (CON) cells in culture. Quantification of invading cell density revealed that shβ2M-expressing cells invaded comparably to non-transduced cells, and calpain 1 silencing had no effect (Fig. 4b). In contrast, silencing of calpain 2 significantly reduced the number of invading cells (Fig. 4b) and the length of invading structures (Fig. 4c). Western blot analyses confirmed successful knockdown of calpains 1 and 2 (Fig. 4d), but also revealed a reproducible upregulation of calpain 2 with calpain 1 knockdown, suggesting calpain 2 may be compensating. To rule out non-specific effects of calpain 2 shRNA, multiple sequences were tested. Compared to ECs expressing shβ2M, shCalp2-2, shCalp2-3 and shCalp2-4 silencing significantly decreased invasion responses (Supplemental Figure 3A). Western blot analyses of lysates from invading cultures revealed successful silencing of β2M and calpain 2 expression in invading cultures (Supplemental Figure 3B). Invasion distance was partially reduced in shCalp2 treatment (Supplemental Figure 3C). Photographs of cultures (Supplemental Figure 3D) revealed a thin sprout morphology with calpain 2 silencing (arrows). These data indicate calpain silencing decreased sprouting responses and are consistent with results with pharmacological calpain inhibition shown in Fig. 3.

### MT1-MMP activation is not extensively altered by pro-angiogenic factors

**Fig. 5:**
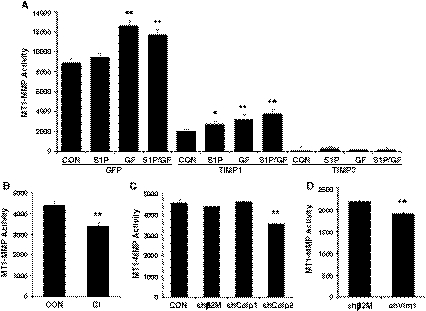
Calpain inhibition and vimentin silencing modestly inhibit MT1-MMP activation. **a** Optimization of conditions to quantify MT1-MMP activation. Stable endothelial cell lines expressing GFP, TIMP-1 and TIMP-3 were generated using recombinant lentiviruses and were allowed to invade for 6 h in the presence of no treatment (CON), 1 μM S1P (S1P), 40 ng/ml VEGF and bFGF (GF) or S1P + GF. Lysates were analyzed using MT1-MMP fluorescence activation assays (see “Materials and methods” section). **b** Quantification of MT1-MMP activity in cultures established using TIMP-1 conditioned medium. Invading cultures (6 h) were treated with vehicle (CON) or CI (100 μM). Lysates from 3D invading cultures (6 h) were prepared **c** from ECs expressing no shRNA (CON) or shRNA directed β2M (shβ2M), calpain 1 (shCalp1) and calpain 2 (shCalp2) and **d** shβ2M and shVim1. Lysates were combined with TIMP-1 conditioned medium and quantified in MT1-MMP activation assays as shown in **a**. All data were expressed as relative MT1-MMP activity and were obtained by performing three replicates per treatment with six collagen matrices collected for each replicate. Data in all panels represent average values ± SD. *P < 0.05, **P < 0.01 versus control by Student’s *t* test

Thus far, our data demonstrate that calpain inhibition decreased sprout density, lumen formation and sprout length, and overall limited the ability of ECs to invade collagen matrices. MT1-MMP is a transmembrane metalloproteinase that cleaves extracellular matrix proteins and mediates sprouting events and lumen formation during endothelial morphogenesis in 3D matrices [26–31]. To test whether calpain and vimentin activate MT1-MMP, MT1-MMP activation assays were performed [34]. Extracts isolated from invading cultures were combined with a fluorogenic peptide sensitive to MT1-MMP cleavage. Because this substrate is also sensitive to cleavage by MMP-1, MMP-2, MMP-7, MMP-8, MMP-12 and MMP-13, various inhibitors were included to rule out a contribution of soluble MMPs to the overall signal. Cells were transduced with lentiviruses expressing GFP control, TIMP-1, and TIMP-3 and allowed to invade collagen matrices with no stimulation (CON), S1P (1 μM), GF (40 ng/ml each VEGF and bFGF) and S1P + GF (Fig. 5a). TIMP-1 expression decreased substrate cleavage compared to GFP-expressing cells, due to inhibition of soluble MMPs. A complete blockade of activity was seen with TIMP-3 expression, fitting with inhibition of all MMPs. The data in the TIMP-1 group are indicative of membrane-associated MMP activity, because TIMP-1 neutralizes soluble MMPs [37]. Importantly, invasion is unaffected by TIMP-1 [38]. Compared to control (CON), S1P and GF slightly increased MT1-MMP activation (Fig. 5A, TIMP-1 treatment). Combined S1P and GF treatment resulted in maximal MT1-MMP activation. In the presence of TIMP-1, MT1-MMP activation was slightly, but significantly, decreased by pretreatment with CI (Fig. 5b), calpain 2 silencing (Fig. 5c), and vimentin knockdown (Fig. 5d). Thus, silencing of calpain and vimentin only modestly decreased MT1-MMP activation.

### S1P stimulated calpain-dependent membrane translocation of MT1-MMP

MT1-MMP is required for endothelial cell invasion [27, 28, 30]. Because calpain inhibition and vimentin silencing significantly decreased invasion responses (Figs. 1, 3) but only marginally reduced MT1-MMP activation (Fig. 5b–d), an alternative mechanism is likely responsible for controlling invasion. Beliveau et al. reported S1P stimulated MT1-MMP membrane translocation [39], and our data show MT1-MMP-GFP translocated to the cell membrane in response to S1P stimulation (Supplemental Figure 4A, white arrowhead), but not following GF stimulation. Data were confirmed in wild type HUVEC using MT1-MMP antibodies (Supplemental Figure 4B) and show that S1P stimulated membrane translocation of endogenous MT1-MMP in ECs.

**Fig. 6:**
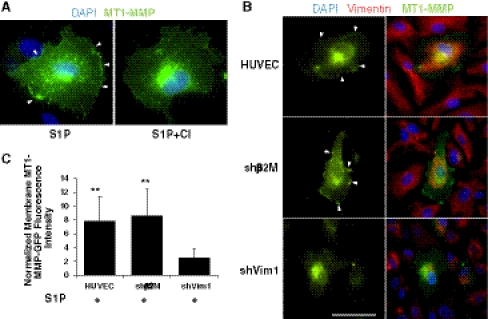
S1P stimulated membrane translocation of MT1-MMP is calpain- and vimentin-dependent. **a** Calpain inhibition blocked S1P-stimulated MT1-MMP membrane translocation. ECs were transfected with MT1-MMP-GFP and seeded overnight on cover slips. Cells were pretreated with vehicle (S1P) or 100 μM CI for 30 min prior to adding S1P for 1 h. Cells were fixed in paraformaldehyde, counterstained with DAPI, mounted and imaged. Arrowheads indicate MT1-MMP-GFP localization to the membrane. **b** Silencing vimentin decreased MT1-MMP membrane translocation. ECs expressing shβ2M (CON) or shVim1 were transiently transfected with MT1-MMP-GFP, seeded on coverslips overnight and treated with 1 μM S1P for 1 h. Following paraformaldehyde fixation, cells were additionally counterstained for vimentin (*red*). *Arrowheads* indicate MT1-MMP-GFP localization to the membrane. *Bar* = 50 μm. **c** Quantification of images shown in **b**. Twenty-five cells in each group were analyzed as described in the “Materials and methods” section. **P < 0.01 versus shVim1 by Student’s *t* test

We next tested whether calpain inhibition altered membrane translocation of MT1-MMP. Pretreating cells with CI decreased MT1-MMP-GFP membrane translocation in response to S1P (Fig. 6a), supporting that calpains are required for MT1-MMP-GFP membrane translocation. These results were confirmed in ECs expressing shCalp2-2 (data not shown). Vimentin knockdown also blocked MT1-MMP translocation to the membrane (Fig. 6b). MT1-MMP fused to green fluorescent protein (MT1-MMP-GFP) translocated to the membrane following S1P stimulation in non-transfected endothelial cells (HUVEC) and ECs expressing shβ2M (white arrowheads), but no membrane localization of MT1-MMP-GFP was seen in vimentin knockdown cells (shVim1). Quantification of MT1-MMP membrane localization showed a significant reduction with shVim1 expression (Fig. 6c). These data were confirmed in cells transiently transfected with vectors encoding MT1-MMP fused to a C-terminal red fluorescent protein (Supplemental Figure 5). These data show that calpain activation and vimentin expression are required for successful S1P-induced membrane translocation of MT1-MMP, and to a lesser extent, MT1-MMP activation.

**Fig. 7:**
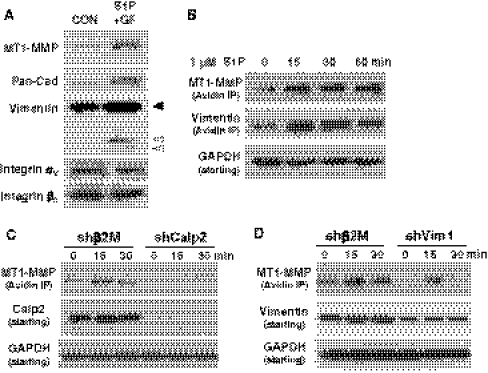
Pro-angiogenic factor-stimulated MT1-MMP membrane translocation is dependent on calpain activation and vimentin. **a** Isolated membrane fractions of 3D cultures were prepared using ultracentrifugation. ECs were allowed to invade in the presence of S1P + GF or nothing (CON) for 3 h. Samples were probed for MT1-MMP, Pan-Cadherin (Pan-Cad), vimentin, and αv and β3 integrin subunits using Western blot analyses. **b** S1P increased MT1-MMP membrane translocation. Cell surface biotinylation assays [34] were utilized for ECs treated with 1 μM S1P for 0, 15, 30 and 60 min. Extracts were incubated with avidin-Sepharose, and eluates were immunoblotted and probed with antisera specific to MT1-MMP and vimentin. Starting material was probed with GAPDH-specific antisera. **c**, **d** Cell surface biotinylation assays were conducted as in **b** with ECs expressing shβ2M and shCalp2 (**c**) and shβ2M and shVim1 (**d**). Cells were treated with S1P for 0, 15 and 30 min and surface labeled. Eluates were probed with antisera directed to MT1-MMP and starting material was probed with antibodies directed to GAPDH, calpain 2 (**c**) and vimentin (**d**)

To provide biochemical evidence for MT1-MMP membrane localization, membrane fractions were isolated from 3D cultures (Fig. 7a). Compared to no treatment (CON), S1P + GF stimulation enhanced MT1-MMP levels in isolated membrane fractions. Probing with Pan-cadherin antisera revealed an increase in cadherin membrane translocation, consistent with a previous report that S1P strengthens adherens junction formation [40]. Interestingly, higher levels of full length vimentin (black arrowhead) and lower molecular weight vimentin fragments (white arrowheads) were also detectable in cultures treated with S1P + GF compared to CON. The αv and β3 integrin subunits levels were similar in both treatment groups and served as loading controls. In Supplemental Figure 6, fractions were probed for known cytosolic and membrane proteins to confirm successful isolation of membrane fractions.

These membrane fractionation studies were reinforced by cell surface biotinylation assays (Fig. 7b). Because S1P predominantly enhanced MT1-MMP membrane translocation (Supplemental Figure 4), these studies were performed with S1P alone. Increased MT1-MMP membrane translocation was observed 15 min after S1P treatment and remained elevated for 1 h (Fig. 7b). Like MT1-MMP, vimentin association with biotinylated surface proteins, increased 15 min after S1P treatment and decreased somewhat at 30 and 60 min (Fig. 7b). We observed this to be full-length vimentin (60 kDa). Calpain 2 silencing decreased MT1-MMP surface biotinylation following S1P treatment (Fig. 7c), compared to shβ2M silencing, where S1P treatment for 15 and 30 min induced MT1-MMP surface biotinylation. Cells expressing shCalp2 lacked induction of surface labeled MT1-MMP, and interestingly, baseline levels at time zero were reduced, as well (Fig. 7c). Identical experiments were conducted with ECs expressing shVim1, where vimentin knockdown was observed with shVim1 expression compared to shβ2M control (Fig. 7d). Like shCalp2 treatment, we observed decreased amounts of surface biotinylated MT1-MMP after S1P stimulation in cells expressing shVim compared to shβ2M controls. These data reinforce that calpain activation and vimentin are required for successful surface translocation of MT1-MMP, which mediates extracellular matrix proteolysis during endothelial sprouting events.

### S1P stimulated vimentin localization to the plasma membrane

**Fig. 8:**
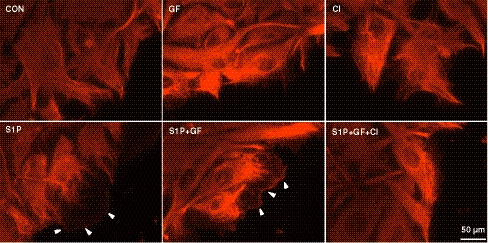
Vimentin localization to the cell periphery requires calpain activation. Cells were seeded on collagen coated coverslips overnight. Monolayers were wounded, washed twice with M199, and allowed to recover for 2 h. In CI groups, cells were pretreated for 30 min (1.5 h post-wounding) prior to S1P + GF treatment. Cells were treated as indicated with nothing (CON), 1 μM S1P, GF (40 ng/ml VEGF and bFGF), S1P + GF, CI, and S1P + GF + CI for 30 min. Immunofluorescence analysis was performed following methanol fixation using antibodies directed to vimentin. *White arrowheads* indicate vimentin localization to the plasma membrane. Note, similar membrane localization was not seen with paraformaldehyde fixation (Fig. 6B)

We next tested whether S1P and GF altered vimentin localization. Vimentin localization was enhanced at the wounded edge of an endothelial monolayer following stimulation with S1P (white arrowheads, Fig. 8), while no localization of vimentin at the membrane was seen in CON or GF treatment. Treatment with S1P + GF appeared similar to S1P alone. CI treatment significantly reduced vimentin localization to the membrane in response to S1P + GF (S1P + GF + CI). These data show that like MT1-MMP translocation to the plasma membrane, vimentin membrane translocation was stimulated by S1P. This is a significant departure from the normal distribution of vimentin which is arranged in polymerized filaments in resting cells.

### Vimentin complexed with MT1-MMP

**Fig. 9:**
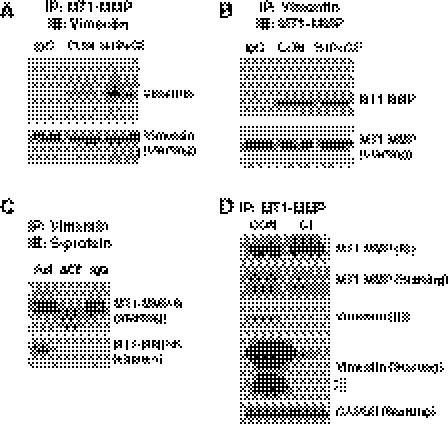
Vimentin complexed with MT1-MMP in 3D invading EC cultures. Experiments in **a**, **b**, **d** were conducted with 3D endothelial cultures at 6 h of invasion. **a** Detergent lysates were immunoprecipitated with MT1-MMP-specific or IgG control antisera (IgG). Samples were probed with antisera directed to vimentin. **b** Reverse immunoprecipitations were performed by combining cleared lysates with vimentin-specific or IgG control antisera. Eluates were probed with MT1-MMP-specific antisera. **c** HEK293 cells were transfected with full length (Full) or cytoplasmic deletions (∆CT) of MT1-MMP containing a C-terminal S-tag. Detergent extracts were cleared and immunoprecipitated with vimentin-specific antisera. Eluates and starting material were probed with S-tag-specific antibodies. An IgG control was included for cells expressing Full length MT1-MMP constructs. **d** Prior to placing endothelial cells on collagen matrices to initiate invasion, cells were treated with DMSO (CON) or 100 μM CI for 30 min in solution. Detergent extracts of 6 h invading 3D cultures were probed with MT1-MMP- and vimentin-specific antisera. Starting material was probed with antisera specific to MT1-MMP, vimentin, and GAPDH

Our data from biochemical and immunofluorescence studies indicated that, like MT1-MMP, vimentin localized to the plasma membrane in response to S1P. To test whether vimentin complexed with MT1-MMP, immunoprecipitations were performed with lysates from 3D invasion cultures. Endothelial cells were seeded on collagen matrices in the absence (CON) or presence of S1P + GF prior to performing immunoprecipitations with MT1-MMP and vimentin antisera. Increased amounts of vimentin immunoprecipitated with MT1-MMP antisera following S1P + GF stimulation (Fig. 9a). MT1-MMP also immunoprecipitated with vimentin antisera (Fig. 9b), although no increase was seen with S1P + GF treatment, possibly because vimentin is an abundant protein and likely only a fraction of total vimentin is captured in reactions. No association between MT1-MMP or vimentin were observed with IgG controls. Thus, a complex containing MT1-MMP and vimentin formed in 3D endothelial cultures.

To test whether vimentin bound to the cytoplasmic tail of MT1-MMP, immunoprecipitations were performed with full length (Full) and cytoplasmic tail deletions (∆CT) of MT1-MMP that expressed a cytoplasmic S-tag (Fig. 9c). Full length, but not ∆CT, MT1-MMP constructs associated with vimentin, and no complex formation was seen with IgG control antibodies, indicating that vimentin associated with the cytoplasmic tail of MT1-MMP.

To determine if calpain inhibition interfered with formation of vimentin-MT1-MMP complexes, lysates were prepared from 3D invading cultures and immunoprecipitated with MT1-MMP-specific antisera. Equal amounts of MT1-MMP were detectable in immunoprecipitates and present in starting material (Fig. 9d). However, in the presence of CI, vimentin did not associate with MT1-MMP (Fig. 9d). Interestingly, probing for detectable amounts of vimentin in the starting material revealed a significant decrease with CI. These data support that calpain cleavage liberated detergent soluble vimentin (which was detectable in the starting material used for immunoprecipitations) and this intracellular pool of soluble vimentin complexed with MT1-MMP.

**Fig. 10:**
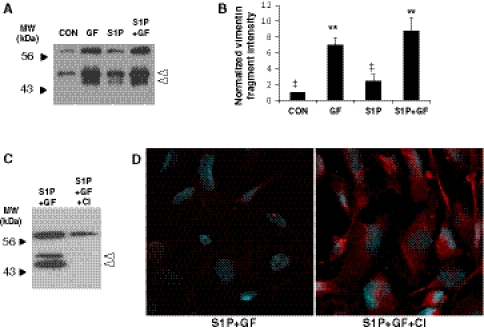
Pro-angiogenic factors stimulated calpain-dependent liberation of detergent-soluble vimentin. **a** ECs were allowed to invade for 6 h. Plates were placed on ice, and wells were washed with 200 μl of cold PBS. Cells were treated with 1% NP-40, 0.05% Na deoxycholate in Hepes Buffered saline, pH 7.4 (40 μl per well). Cleared supernatants were used for Western blot analyses using vimentin-specific antisera. **b** Quantification of intensities of vimentin cleavage products with treatment conditions. Data are derived by averaging band intensities from three independent experiments. **P < 0.01 versus CON; ^‡^ *P* < 0.05 versus all other treatments by Student’s *t* test. **c** ECs were allowed to invade for 6 h in the presence of S1P and GF. Cells were pre-treated with DMSO (S1P + GF) or 10 μM CI. Samples were processed as described in **a** and probed for vimentin using Western blot analyses. **d** Following collection of supernatants analyzed in **c**, cultures were fixed in paraformaldehyde and probed for vimentin using immunofluorescence. *Red* staining indicates vimentin and *blue*, DAPI

Calpain cleaves the N-terminus of vimentin [18]. An intact N-terminal rod of vimentin is required to form polymerized intermediate filaments [20], and vimentin fragments generated following calpain cleavage fail to polymerize [41]. Polymerized intermediate filaments are resistant to detergent solubilization. Based on these properties of vimentin, we tested whether pro-angiogenic factors altered the state of vimentin polymerization by extracting with a detergent solution. Supernatants of 6 h invading cells were collected after detergent solubilization and analyzed by Western blotting. Compared to no treatment (CON), soluble vimentin levels were enhanced slightly with S1P treatment, but more effectively enhanced with GF treatment (Fig. 10a). Combining S1P + GF treatment was not significantly higher than GF alone, suggesting GF treatment predominantly stimulated vimentin cleavage. Data were quantified in Fig. 10b. Thus, GF induced vimentin cleavage and enhanced soluble vimentin fragments in invading ECs. In separate experiments, pre-treatment of 3D invading cultures with CI decreased detectable amounts of soluble vimentin compared to treatment with S1P + GF alone (Fig. 10c). Fitting with this observation, immunofluorescence staining indicated more intact (or detergent insoluble) vimentin was present with calpain inhibition (Fig. 10d). In agreement with these results, calpain 2 silencing decreased vimentin cleavage in whole cell lysates and detergent lysates (Supplemental Figures 7A and 7B, respectively).

Overall, these data show that GFs predominantly activate calpains, which (presumably through cleavage) stimulate vimentin depolymerization and liberation of detergent soluble vimentin. We find here for the first time that soluble vimentin complexed with the cytoplasmic tail of MT1-MMP and facilitated S1P-induced MT1-MMP membrane translocation, which is required for endothelial cell invasion.

## Discussion

We report here a mechanism to control endothelial sprouting in 3D collagen matrices, which mimics activation of the angiogenic switch. Calpain activation altered the polymerization state of vimentin, which complexed with MT1-MMP to accomplish proper membrane localization of MT1-MMP, which facilitated collagen degradation to allow successful sprouting responses, as others have reported requires MT1-MMP [26–28, 30]. The results presented here show for the first time that calpain-dependent depolymerization of intermediate filaments directs successful membrane localization of MT1-MMP and results in endothelial cell sprouting responses.

Our results are the first to demonstrate an endothelial-specific requirement for vimentin in controlling angiogenic responses. While there has been some evidence implicating vimentin in regulating angiogenic and vasculogenic responses [10, 11, 42], a direct requirement for vimentin in sprouting ECs has not been reported. Vimentin is an intermediate filament (IF) that is involved in many important physiological functions, such as the distribution of organelles, signal transduction, cell polarity and gene regulation [43–45]. To our knowledge, this is the first report that detergent soluble vimentin complexes with MT1-MMP, and our data are consistent with vimentin liberation by active calpains. Calpains cleave the N-terminal region, or head of vimentin [18, 19]. The head region is required for assembly of IF networks [41, 46]. Headless vimentin cannot polymerize or assemble into filaments, but can assemble into monomers, dimers or tetramers [47]. Tetrameric vimentin complexes tend to be homogenous and increase in the presence of physiological salts and pH 7.5 [48]. Further, headless vimentin can prevent full-length vimentin from polymerizing, and can partially disrupt polymerized networks comprised of full-length vimentin [48]. The soluble vimentin we detect is comprised of full length (60 kDa) and truncated vimentin (43–50 kDa), indicating that the soluble vimentin pool generated by calpain cleavage may be comprised of mixed complexes of cleaved and soluble vimentin, which do not assemble into regular IF networks, but may be available for associating with MT1-MMP to facilitate matrix proteolysis and endothelial sprouting. Although we can only detect full length vimentin associated with MT1-MMP in immunoprecipitation experiments, this may be the result of experimental binding conditions and overnight incubations. Thus, we cannot rule out binding of headless vimentin to MT1-MMP. One possibility we have not addressed here is that other signals, such as vimentin phosphorylation, which also induces depolymerization [49], may be necessary to completely disrupt polymerized vimentin networks.

We show that calpain activation, vimentin cleavage, and increased vimentin solubility following GF stimulation couple with MT1-MMP membrane translocation that is driven by S1P to facilitate sprouting responses. We observed that MT1-MMP activation was not controlled by calpain activation or the presence of vimentin because vimentin knockdown and calpain inhibition only modestly blocked MT1 activation, yet significantly block invasion. Thus, localization of MT1-MMP was not direct by calpains. We observed that S1P, but not GF, stimulated localization of MT1-MMP to the plasma membrane using multiple approaches. The ability of S1P to stimulate MT1-MMP surface translocation may explain why S1P combines with either GF or WSS to stimulate invasion responses [33, 50]. GF and WSS activate calpains [22, 51], but our data suggest that global calpain activation is not sufficient to explain sprouting responses. Neither GF nor WSS treatment alone stimulated invasion (Supplemental Figure 1, [50]). Thus, calpain activation, and subsequent vimentin cleavage are not sufficient for initiating sprouting responses, but must be combined with proper membrane localization of MT1-MMP, which is driven by S1P. Thus, integration of multiple extracellular signals are needed to activate the angiogenic switch.

We report that vimentin complexed with the cytoplasmic tail of MT1-MMP. This region of MT1-MMP is involved in endocytic recycling and activation of MT1-MMP [52]. S1P stimulated membrane translocation of MT1-MMP that is dependent on phosphorylation of Tyr^573^ [39]. We showed here that S1P-induced MT1-MMP membrane translocation required calpain activation and vimentin. In addition, pro-angiogenic factors increased the amount of soluble vimentin available to complex with MT1-MMP. Silencing vimentin and calpain significantly reduced MT1-MMP surface translocation in response to S1P. Our data support a model in which pro-angiogenic signals activated calpain, which cleaved and depolymerized vimentin; This pool of soluble vimentin complexed with the cytoplasmic tail of MT1-MMP and aided MT1-MMP cell surface translocation. Vimentin fragments generated by calpain cleavage have been reported to complex with and protect Erk1/2 from dephosphorylation to facilitate neuronal repair [53, 54], providing some support for the possibility that complex formation by vimentin might regulate MT1-MMP phosphorylation levels. Whether vimentin availability affects phosphorylation of MT1-MMP remains to be tested. In addition, probing the specific binding interactions between MT1-MMP and vimentin is warranted.

MT1-MMP has been established as a key mediator of EC sprouting processes [28, 30]. Because MT1-MMP is tethered to the membrane, it is perfectly suited to mediate controlled extracellular matrix proteolysis in close proximity to the cell surface [26, 30, 31, 55]. Consequently, the signals that regulate membrane localization of MT1-MMP are critical to understand. Calpains have previously been implicated in controlling angiogenic responses, and we have found recently calpain is necessary for MT1-MMP surface translocation in a model of wall shear stress and S1P-induced invasion [56]. Senger et al. previously reported that moderate inhibition of calpain led to normalization of neovascularization responses in multiple models of pathological angiogenesis [25, 57]. One potential explanation for those data, supported by our findings here, is that moderate calpain inhibition also partially limited MT1-MMP-mediated proteolysis and new blood vessel growth, allowing slightly less robust and perhaps more controlled angiogenic responses to occur. This inhibition ultimately resulted in a more normalized vasculature. Thus, our data support the conclusions of Senger et al., who showed that manipulation of calpain activity is a viable strategy for normalizing pathological angiogenesis [25, 57], as is controlling surface translocation of MT1-MMP.

Here, we demonstrate that vimentin is associated with the cell membrane in invading ECs, consistent with a previous report [58]. Our data suggest that vimentin participates in assembling a molecular complex with MT1-MMP to coordinate EC sprout extension and invasion. Vimentin localization was found to increase in anterior portions of ECs traversing filters in Boyden chamber assays and in the developing retinal vasculature [59]. Further, caveolin co-purified with intermediate filaments in ECs, and this interaction required Tyr^14^ [59]. Interestingly, MT1-MMP is enriched in caveolae [60], but MT1-MMP does not contain a consensus binding site for caveolin. Thus, our data suggest that vimentin may provide a molecular link between caveolin and MT1-MMP. Vimentin also complexed with the α2β1 integrin and co-localized with α2β1 in focal adhesion complexes [61]. Davis et al. have shown α2β1 is part of a signaling complex that contains MT1-MMP and controls EC tubulogenesis and lumen formation [62], and the α2β1 integrin mediates EC invasion in type I collagen matrices [38]. We find that vimentin is present in membrane preparations, which has previously been reported [58], raising the possibility that vimentin participates in assembling molecular complexes composed of caveolin Tyr^14^, MT1-MMP, the α2β1 integrin and other surface molecules to coordinate EC sprout extension and invasion. It is tempting to speculate that integrin recognition of collagen, a pro-morphogenic substrate for ECs [63], initiates assembly of a molecular complex composed of α2β1, MT1-MMP, and caveolin Tyr^14^ at the cell surface that is stabilized by vimentin to facilitate endothelial morphogenesis and sprouting events.

In summary, our data define a pathway downstream of pro-angiogenic factors for calpain activation, vimentin cleavage and depolymerization, and MT1-MMP surface translocation that controls the initiation of endothelial cell sprout formation. We find that calpain and vimentin are required to regulate proper membrane translocation of MT1-MMP, explaining the ability of calpain inhibition and vimentin silencing to block endothelial sprouting events. Because these molecules are universally expressed (and in the case of vimentin, upregulated) in malignant disease, this pathway may also regulate invasive, metastatic cell behavior. These data for the first time demonstrate a functional link between calpain activation, vimentin cleavage, vimentin reorganization, and membrane translocation of MT1-MMP in EC sprout initiation.

## Electronic supplementary material

Below is the link to the electronic supplementary material.

Supplementary material 1 (DOC 8753 kb)
