# Supplementary material for: Calpain-mediated vimentin cleavage occurs upstream of MT1-MMP membrane translocation to facilitate endothelial sprout initiation
Source: Angiogenesis. 2012 Mar 11;15(2):287–303. doi: 10.1007/s10456-012-9262-4 (PMC3338915; doi:10.1007/s10456-012-9262-4)
Supplement: Supplementary file 1 — Supplementary material 1 (DOC 8753 kb) [file 10456_2012_9262_MOESM1_ESM.doc]

**Supplemental Figure 1.** **S1P and GF combine to stimulate endothelial sprouting in 3-D collagen matrices.** Cells were allowed to invade in the presence of no treatment (CON), 1 µM S1P (S1P), 40ng/ml VEGF and bFGF (GF) or S1P+GF (S1P/GF). Invasion responses were quantified after 24 hours of invasion. Data shown are average values from two independent experiments (n=6; 3 wells per experiment) ± SD.

**Supplemental Figure 2.** **Calpains are activated by pro-angiogenic stimuli.** (A) Invading cultures were extracted at 3hr and fresh lysates were quantified using Calpain-Glo™ Protease Assay Kit (Promega). (A) Calpain activity in the presence of no treatment (CON), 1 µM S1P (S1P), 40ng/ml VEGF and bFGF (GF) or S1P+GF. (B) Calpain activity in 3 hour invading cultures stimulated with S1P+GF were pretreated for 30 minutes with no treatment (CON) or calpain inhibitor-III (CI, 31.6µM) prior to initiating invasion. All data are expressed as relative units ± SD and are obtained by performing 3 replicates (n=6 wells each) per treatment. **P<0.01 versus all other groups by ANOVA.

**Supplemental Figure 3.** **Calpain 2 knockdown significantly reduced invasion.** ECs were transduced with lentiviruses delivering shRNA directed to beta 2 microglobulin (shβ2M; negative control) and calpain 2 (shCalp2-2, -3 and -4). Stable cell lines were selected with puromycin (0.2ug/ml) for two weeks prior to testing in invasion assays. (A) Quantification of invasion density (16 hours). Representative experiment (n=3) presenting average values ± SD from 4 random fields. (B) Western blot analyses of invading cell lysates (24 hours) were conducted to confirm successful protein silencing. GAPDH served as a loading control. (C) Distances (in micrometers) from monolayer to leading edge of invading structures (n=100 cells). Data represent mean ± SD. **P<0.01 vs. control, Student’s t-test. (D) Representative photographs of a side view of invading cells in control (shβ2M) and shCalp2-2 expressing cells. Arrows indicate altered structures observed with shCalp2-2 expression; arrowheads indicate original monolayer. Scale bar, 50 µm.

**Supplemental Figure 4. S1P stimulated membrane translocation of MT1-MMP.** (A) HUVECs were transiently transfected with vectors expressing MT1-MMP-GFP constructs before being seeded overnight on glass coverslips. The next day, growth media was removed and cells were treated with nothing (CON), 1 µM S1P (S1P), 40ng/ml VEGF and bFGF (GF) or S1P+GF. Cells were fixed in paraformaldehyde and counterstained with DAPI (blue signal). (B) Wild-type HUVECs were seeded on coverslips overnight as confluent monolayers. Wound assays were performed and cells were washed twice with M199. Cells were then treated for 3 hours in the presence of no treatment (CON), 1 µM S1P (S1P), 40ng/ml VEGF and bFGF (GF) or S1P+GF. In all panels, cells were fixed in paraformaldehyde, stained with MT1-MMP-specific antisera detected by secondary antibodies conjugated to Alexa 488, mounted and imaged. White arrowheads indicate MT1-MMP membrane localization. Upper panels show endothelial cells adjacent to the wound edge, and lower panels show confluent areas.

**Supplemental Figure 5.** **S1P stimulated vimentin-dependent membrane translocation of MT1-MMP.** Silencing vimentin decreased MT1-MMP membrane translocation. ECs expressing shβ2M or shVim1 were transiently transfected with MT1-MMP-RFP, seeded on coverslips overnight and treated with 1µM S1P for 3 hours. Following paraformaldehyde fixation, cells were additionally counterstained for vimentin (green). Arrowheads indicate MT1-MMP-RFP localization to the membrane.

**Supplemental Figure 6.** **Successful separation of membrane and cytoplasmic cellular fractions.**  Isolated membrane and cytoplasmic fractions of 3D cultures were prepared using ultracentrifugation. ECs were allowed to invade in the presence of S1P+GF or nothing (CON) for 3 hours. Samples were probed for connexin 43 (rabbit polyclonal, Sigma-Aldrich), β1 integrin subunit (BD Biosciences), and GAPDH using Western blot analyses.

**Supplemental Figure 7. Calpain 2 knockdown in ECs results in reduced levels of soluble vimentin that correlate with decreased invasion responses.** ECs were transduced with no lentivirus (HUVEC) or recombinant lentiviruses delivering shRNA directed to beta 2 microglobulin (shβ2M), Calpain 1 (shCalp1), Calpain 2 (shCalp2). (A) Western blot analyses of whole cell lysates of invading cells (6 hours) using calpain 2-, vimentin- and tubulin- specific antisera. Black arrowhead indicates total vimentin and white arrowhead indicates vimentin fragments. (B) Western blot analysis of soluble cell lysates of invading cells (6h) using calpain2-, vimentin-, tubulin- and β2M- specific antisera. (C) Quantification of invasion density resulting from shRNA mediated knockdown of beta 2 microglobulin (shβ2M), calpain2 (shCalp2) and HUVEC (control) in this experiment. Data represents average numbers of invading cells per standardized field (n=3 fields, **P<0.01 compared to shβ2M, Students *t*-test. (D) Representative photographs illustrating invasion responses (top-view, upper panels; side-view, lower panels).
